# In the moment social experiences and perceptions of children with social anxiety disorder: A qualitative study

**DOI:** 10.1111/bjc.12393

**Published:** 2022-10-10

**Authors:** Brynjar Halldorsson, Polly Waite, Kate Harvey, Samantha Pearcey, Cathy Creswell

**Affiliations:** ^1^ Department of Experimental Psychology University of Oxford Oxford UK; ^2^ Department of Psychiatry University of Oxford Oxford UK; ^3^ Oxford Health NHS Foundation Trust Oxford UK; ^4^ Department of Psychology Reykjavik University Reykjavik Iceland; ^5^ School of Psychology and Clinical Language Sciences University of Reading Reading UK

**Keywords:** CBT, maintenance mechanisms, qualitative, Social anxiety disorder, treatment

## Abstract

**Objectives:**

Childhood social anxiety disorder (SAD) is a common and disabling condition. General forms of cognitive behavioural treatments have demonstrated poorer efficacy for childhood SAD when compared to other childhood anxiety disorders and further understanding of the psychological factors that contribute to the maintenance of childhood SAD is warranted. Examining the social experiences of children with SAD may help to identify relevant psychological factors and increase our understanding of what keeps childhood SAD going.

**Methods:**

The current study used reflexive thematic analysis to analyse the transcripts of interviews with 12 children aged 8–12 years with SAD who had been interviewed about their ‘in the moment’ social experiences during a social stress induction task. The interview topic guide included factors hypothesized to maintain SAD in adult cognitive models of the disorder.

**Results:**

The interviews revealed both variety and commonalities in the experiences and interpretations of social events in children with SAD, captured in three related main themes: (i) Discomfort being the centre of attention, (ii) (Lack of) awareness of cognitions and (iii) Managing social fears. Findings indicated likely developmental influences on which maintenance mechanisms apply at which point in time.

**Conclusions:**

There is variation in the psychological mechanisms that children with SAD endorse and developmental factors are likely to influence when specific mechanisms are relevant. We now need further studies that take a developmentally informed approach to understand the nature of the association between the factors identified in this study and social anxiety in childhood to inform the development of more effective interventions for childhood SAD.

## Practitioner points

- This study focused on ‘in the moment’ social experiences and perceptions of pre‐adolescent children with social anxiety disorder (SAD) in order to inform a greater understanding of what cognitive and behavioural factors play a role in maintaining SAD in children.
- This qualitative study is, to our knowledge, the first to directly explore how pre‐adolescent children with SAD map onto adult cognitive models of SAD.
- It seems likely that there are developmental influences on which maintenance mechanisms apply at which point in time as only older children in the sample reported safety‐seeking behaviours, negative imagery and post‐event processing.
- Existing treatments for childhood SAD may need to be adapted in order to target the different psychological mechanisms that prevent children with SAD from overcoming their social anxiety.

## BACKGROUND

Social anxiety disorder (SAD) is the most common anxiety disorder, with up to 13% of the population meeting the diagnostic criteria at any point in time (Beesdo et al., 2007; Kessler, Chiu, et al., 2005). The median age of onset of SAD is estimated at 13 years (Kessler, Berglund, et al., 2005), and it is a commonly diagnosed condition in clinical samples of pre‐adolescent children (i.e., 12 years and younger; from here on ‘children’) (Esbjørn et al., 2010; Waite & Creswell, 2014). In the absence of effective treatment, SAD often runs a chronic course with poorer recovery rates than other anxiety problems (Bruce et al., 2005) and in children, results in significant functional impairment across various domains, including academic, social and family functioning (American Psychiatric Association, 2013; Beesdo et al., 2007; Beidel et al., 1999; Schutters et al., 2011). Thus, early detection and effective treatment are essential for children with SAD.

At present, the most frequently used psychological treatment for childhood SAD is multi‐disorder‐focused cognitive behavioural therapy (CBT), that is CBT programmes that can be applied across a range of anxiety disorders (e.g., Kendall & Hedtke, 2006). This is partly driven by the clinical characteristics of child anxiety disorders, in that there is a high level of comorbidity between them. However, there is evidence to suggest that the presence of SAD in the diagnostic spectrum is associated with poorer response to general forms of CBT compared with other anxiety disorders (Evans, Clark et al., 2021; Ginsburg et al., 2011; Hudson et al., 2013). The reasons why children with SAD have worse outcomes than children with other types of anxiety disorders are not clear. It is possible that these treatments do not target psychological maintenance mechanisms that are specific to childhood SAD. For example, it has been suggested that children with SAD have social skills deficits which should be targeted in treatment (Rapee & Spence, 2004; Spence et al., 2000; Spence & Rapee, 2016). Notably, however, evidence for social skills deficits in childhood SAD is mixed (Halldorsson & Creswell, 2017; Pearcey et al., 2020) and approximately 30%–50% of children with SAD still retain their SAD diagnosis after receiving disorder‐specific treatments for childhood SAD that target social skills deficits (Beidel et al., 2000, 2007; Donovan et al., 2015; Öst et al., 2015; Spence et al., 2000).

In contrast, highly effective CBT treatments have been developed specifically for adults with SAD which directly target cognitive and behavioural mechanisms that maintain SAD (Clark et al., 2006; Mörtberg et al., 2007; Stangier et al., 2003), specifically: (i) interpretation biases (including dysfunctional beliefs); (ii) self‐focused attention and self‐monitoring; (iii) misleading internal information (e.g., negative images, body arousal); (iv) safety‐seeking behaviours; and (v) detailed and catastrophic anticipatory and post‐event processing (for further details see Clark, 2005). To date, we know very little about what psychological mechanisms maintain childhood SAD (Halldorsson & Creswell, 2017). Instead, recent conceptualizations of SAD in children have typically focused on development (Ollendick & Benoit, 2012; Ollendick & Hirshfeld‐Becker, 2002; Rapee & Spence, 2004; Spence & Rapee, 2016) rather than maintenance so do not directly inform treatment and its components.

In a recent review to examine whether, or to what extent, the same cognitive and behavioural maintenance processes that occur in adult SAD also apply to childhood SAD, Halldorsson and Creswell (2017) found (albeit limited) evidence that, compared with non‐anxious children, children with SAD have a tendency to interpret social situations as threatening, use safety‐seeking behaviours, focus their attention inwards and engage in negative anticipatory/post‐event processing. However, the review did not identify any studies that examined the other putative maintenance mechanisms: dysfunctional beliefs, negative distorted images and diffused body perception within the context of childhood SAD. Turning to studies examining social anxiety in adolescents, a recent review (Leigh & Clark, 2018) and emerging experimental studies (Leigh et al., 2020, 2021) have provided support for the application of Clark and  (1995) model to this age group.

A clear understanding of the maintenance of SAD in childhood must also consider the possible influence of children's cognitive developmental status. The human brain undergoes significant development throughout the pre‐adolescent period (Burnett et al., 2011; Crone & Van Der Molen, 2007; Sebastian et al., 2010; Supekar et al., 2009), particularly in brain regions implicated in emotion‐ and social‐processing and self‐awareness (Casey et al., 2005). Thus, it is possible that specific processes outlined in the adult SAD cognitive models may not come ‘online’ until children have reached certain developmental stages. For example, children's cognitive capacity to see themselves as others see them may not fully develop until late childhood (Cole et al., 2001). Also, there is evidence to suggest that children and adults process facial emotional expressions differently (Thomas et al., 2001), use distinctive neurocognitive strategies for making self‐referential judgements (Pfeifer et al., 2007), and that children are less effective than adults in analysing the intentionality of other's behaviour and mental states during social interactions (Güroğlu et al., 2009). Furthermore, the role of social‐environmental factors, including the influence of other people (e.g., parents, peers and teachers) on children's developing cognitions, changes markedly throughout development (Cole et al., 1997, 2001). Thus, developing further understanding of the cognitive, behavioural and environmental factors that contribute to the maintenance of SAD and treatment outcomes in psychological treatments, and how they operate across development and among children with SAD, will be critical to enable us to continue to improve treatment outcomes for this population.

Clark (2004) described a sequential approach to empirical research that has been particularly helpful in both identifying adult SAD maintenance mechanisms and devising a highly effective and acceptable psychological treatment. This involves (i) conducting clinical/qualitative interviews to formulate a theory of the processes hypothesized to maintain the disorder, followed by (ii) testing the theory using experiments and prospective longitudinal studies, (iii) developing and evaluating a specialized cognitive intervention that targets the maintenance mechanisms and (iv) disseminating the resulting intervention. Looking to the first stage of this approach, we know surprisingly little about ‘in the moment’ experiences (and pre‐ and post‐event experiences) of children with SAD and how contextual and developmental factors influence these experiences. The limited existing studies have mainly focused on children's negative cognitions, indicating that, compared with non‐anxious children, children with SAD experience more negative thoughts during social‐evaluative tasks (e.g., Spence et al., 1999; Tuschen‐Caffier et al., 2011), but we know little about other cognitive and behavioural ‘in the moment’ processes. Thus, in order to ultimately inform understanding of the maintenance of childhood SAD, we interviewed children with SAD about their experiences during a social stress induction task. Reflexive thematic analysis (Braun et al., 2018) was applied with the aim of increasing our understanding of what cognitive and behavioural factors may play a role in maintaining social anxiety in children.

## METHODS

### Participants

A total of fifteen children aged 8–12 years diagnosed with SAD as their primary presenting problem was invited to participate in the study. Children were not excluded on the basis of comorbid diagnoses to reflect a typical clinical population. Exclusion criteria for the recruiting clinical service were active suicidality or severe self‐harm, significant physical or intellectual impairment and/or diagnosis of autism spectrum disorders. Three families (one child and two parents) declined participation (the child did not give a specific reason; and the parents declined due to time constraints), resulting in a final sample of 12 participants. The concept of information power (Malterud et al., 2016) was used as a guide for ascertaining a sample size for reflexive TA. Information power indicates that the more information relevant to the study aim that a sample holds, the fewer participants are needed. Given the nature of this sample and the challenges in recruiting socially anxious children, it was considered appropriate. Sample size was assessed throughout the analysis, and we agreed as a team that we had reached sufficient information power when we had detailed and sufficiently rich information on how social anxiety presented in this group. Children were recruited (consecutively) following a referral by local health and education service personnel to a specialist anxiety and depression clinic and research centre in the United Kingdom. All children with a diagnosis of SAD that came through the clinic were invited to take part in the study during the recruitment phase of the study.

### Measures

#### Structured diagnostic interviews

Children and adolescents were assigned diagnoses on the basis of semi‐structured diagnostic interviews based on DSM‐IV, with minor amendments to enable diagnoses consistent with DSM‐5 diagnostic criteria. The Anxiety Disorder Interview Schedule for DSM‐IV for children, child and parent versions (ADIS‐C/P; Silverman & Albano, 1996) was used to assess anxiety disorders and behavioural disorders, and the Kiddie Schedule of Affective Disorders and Schizophrenia (K‐SADS; Kaufman et al., 1997) was used to assess depressive disorders. As is conventional with both assessments, the interviews were conducted with the child and their parent/s separately. Reliability for presence or absence of anxiety diagnosis on the ADIS‐C/P was κ = 1.00 and CSR ICC = .93. For the K‐SADS, depression diagnoses were based on the combined information obtained from both interviews, as is standard, and inter‐rater reliability was k = 1.00.

*The Revised Child Anxiety and Depression Scale* (RCADS; Chorpita et al., 2000) was completed separately by parents and children. The RCADS is a 47‐item parent and child report scale which assesses symptoms of separation anxiety disorder, social anxiety disorder, generalized anxiety disorder, panic disorder, obsessive–compulsive disorder and major depressive disorder. Responders rate how often each item applies on a scale of 0 (‘never’) to 3 (‘always’). The RCADS has been shown to have robust psychometric properties in children and young people from 7 to 18 years of age (Chorpita et al., 2005). Internal consistency based on data from the current sample was good (Cronbach's α .91 for parent report and .95 for child report).

*The Liebowitz Social Anxiety Scale for Children and Adolescents* (LSAS‐C/A; Masia‐Warner et al., 1999) was administered to assess children's self‐reported social anxiety symptoms. The self‐report version of the LSAS‐C/A was used and includes 24 items, rated on a scale from 0 ‘none’ to 3 ‘severe’, to assess fear and avoidance of social interaction and performance (Masia‐Warner et al., 1999). A cut‐off score of 22.5 is considered to distinguish between individuals with SAD and normal controls. The LSAS‐C/A has well‐established psychometric properties when administered to children and young people from 7 to 18 years of age (Masia‐Warner et al., 2003) with good internal consistency from the current sample (Cronbach's α = .88 for fear subscale and .89 for avoidance subscale).

#### Social stress task

In order to gain access to ‘in the moment’ social experiences of the participants (rather than relying on recall or hypothetical discussion), the qualitative interviews were conducted in front of a pre‐recorded audience of similar‐aged children. The children in the recording were broadly attentive and neutral in their responses. Participants were told that the audience was pre‐recorded but asked to imagine that they were standing in front of a live audience. To maintain the social‐evaluative nature of the situation, the audience was filmed in a classroom setting with children of a similar age to the participants and the video was projected life‐size (playing audio of a busy classroom) to create a sense of a live audience (in line with similar recordings; e.g., Westenberg et al., 2009).

#### Indicative topic guide

A topic guide (Appendix S1) was developed to explore children's experiences relating to mechanisms hypothesized to maintain SAD in adult cognitive models of the disorder during an *in vivo* social stress task. The topic guide questions were based on the literature (in particular Clark and Wells (1995) and Rapee and Heimberg (1997) cognitive models) and discussions with experts and explored children's tendency to engage in (or experience): pre‐ and post‐event processing; self‐focused attention and self‐monitoring; negative images; and safety‐seeking behaviours. The topic guide was used flexibly, allowing for variation in the order and wording of questions and ensuring children had the opportunity to discuss issues that departed from the prepared areas of questioning. The topic guide was modified iteratively, as the interviews and concurrent data analysis proceeded, to incorporate new information and focus progressively on themes.

In order to help children to understand the questioning and be able to express themselves, several items and prompts were used. For example, when children were asked about their cognitions, they were encouraged to write down or illustrate their thoughts using ‘thought bubbles’; when expressing negative imagery, they were given access to paper and coloured pens to illustrate the images. To identify bodily symptoms of anxiety, children were shown pictures of people experiencing common physical symptoms of anxiety as prompts (e.g., a person holding their tummy). Furthermore, complicated constructs such as self‐focused attention were explained using several prompts; a torch (pointed towards the pre‐recorded audience and the self) and printed pictures (explaining the difference between first person and third person perspective). Interviews lasted between 20 and 40 min (with the video running most of the time on a loop). They were audio and video recorded, and all content was transcribed in full and anonymized at the point of transcription. Observations from the video recording and the researcher's fieldnotes contributed to the dataset.

### Procedure

Parents of all participants gave written informed consent and children provided assent. All procedures received University and National Health Service ethical approval (reference number removed for blind review). Once consent/assent was gained and eligibility confirmed, participants were invited to attend the research session. Upon arrival, children and their parent/s completed the questionnaires described below. They were then directed into the laboratory, and the child was informed that they would shortly see a video projected onto a wall of a pre‐recorded audience (of similar age children), which would likely trigger feelings of anxiety and their task involved answering questions whilst the video was playing. Once children had settled in, parents were asked to leave the room and the interview began. Children were encouraged to keep watching the video whilst answering the questions. Participants were told that they could stop the interview at any point. In order to keep the flow of the video going, it was not stopped whilst children's answers were elicited. Participants were given the opportunity for discussion once the video ended. One week later, participants were invited to answer further questions (via phone or in person and lasting approximately 5–10 min) focussing on post‐event processing but without the pre‐recorded audience. Participants received a voucher for their help and time with the study. The topic guide and social stress task procedures were piloted with three children and amendments made based on their feedback.

### Data analysis

The consolidated criteria for reporting qualitative research (Tong et al., 2007) checklist were followed. We used Nvivo V12 to support reflexive thematic analysis of participant transcripts (Tong et al., 2007). Reflexive thematic analysis was chosen because it is a theoretically flexible method of analysis (Braun et al., 2018; Braun & Clarke, 2013), which allows a focus on both the identification of commonalities and variations in children's social experiences. Coding involved both an inductive and deductive approach as our aim was both on theory building (inductive) and testing an existing theory (deductive; e.g., maintenance mechanisms identified in the adult models). Data from both interviews (those focused on in the moment social experiences and those focused on post‐event processing) were analysed together as one broad dataset. The analysis followed Braun and Clarke (2006, 2013) and Braun et al. (2018) six‐phase approach: (i) Familiarization with the data, (ii) Coding; (iii) Generating initial themes, (iv) Reviewing themes, (v) Defining and naming themes and (vi) Writing up. Each phase builds on the previous, with movement back and forth between phases (Braun & Clarke, 2006, 2013).

The first author, a clinical psychologist with experience in delivering CBT for SAD, completed all interviews, transcribed the data, led the analysis and discussed each stage in the process with KH (a qualitative researcher with no experience in treating children with SAD), PW and CC (both clinical psychologists experienced in treating childhood SAD). During discussions, the authors encouraged each other to clarify and refine their interpretations–with the aim of optimizing the rigour and quality of the analysis. Initial stages involved developing a set of themes to capture the children's experiences of social anxiety. The team then moved to a more deductive approach, where the authors attempted to explore how children's experiences mapped onto adult models of SAD. This led to further refining of the themes, which were again discussed by the whole research team, thereby increasing our confidence in the robustness of our thematic structure to capture the range of experience described by the children in the study.

## RESULTS

### Demographic information

Child characteristics, clinical severity ratings and child‐ and parent‐reported anxiety are reported in Table 1.

**TABLE 1:** Child characteristics, clinical severity and levels of social anxiety

ID	Sex	Age	Ethnicity	ADIS CSR for SAD	Comorbid anxiety disorder (ADIS CSR)	Child RCADS total (t‐scores)	Parent RCADS total (t‐scores)	Child LSAS‐C/A total
AA	Male	12	White	7	GAD (4)	62	>80	86
BB	Female	11	White	7	GAD (5); PHO (5); SEP (4)	52	70	93
CC	Male	11	White	6	GAD (5)	61	>80	112
DD	Male	12	White	6	SEP (4)	58	>80	86
EE	Female	12	White	6	GAD (4)	52	64	99
FF	Female	11	White	6	PHO (5); GAD (5); SEP (4); PAN (4)	49	75	60
GG	Female	9	White	4	–	36	60	25
HH	Female	12	White	7	GAD (6); SEP (6); MDD (6)	70	>80	110
II	Female	12	White	5	–	76	78	89
JJ	Female	11	White	5	–	45	64	77
KK	Female	10	White	6	SEP (6); PHO (6)	40	65	45
LL	Male	8	White	4	–	45	58	103

Abbreviations: CSR, clinical severity rating (0–8); GAD, generalized anxiety disorder; LSAS, Liebowitz social anxiety scale for children and adolescents; MDD, major depressive disorder; PAN, panic disorder; PHO, specific phobia; RCADS, revised children's anxiety and depression scale; SEP, separation anxiety disorder.

### Qualitative results

When asked, most children reported (and appeared) feeling anxious before and after standing in front of the pre‐recorded audience and expressed relief once the video‐recorded audience was turned off, suggesting that the projection of a video‐recorded audience successfully induced anxiety. Notably, a subset of children experienced debilitating levels of anxiety during the interview. This was evident from their behaviour and body language, such as covering their faces with their hair, fiddling with their hands, avoiding looking at the screen, crying, whispering or in some cases not speaking at all. Three interviews (participants EE, JJ and LL) had to be stopped before they could be completed, due to children's distress.

The interviews revealed both variety and commonalities in the experiences and interpretations of social events in children with SAD, captured within three main themes. The first theme ‘Discomfort being the centre of attention’ contained three, related, subthemes (‘I will do something wrong’; ‘I am being judged’ and; ‘Sense of being stared at’) and concerned *why* children with SAD find social situations threatening. The second theme, ‘(Lack of) awareness of cognitions’, concerned children's ability to identify (or articulate) their cognitions, with participants reflecting one of four main types: those who were able to report their cognitions (‘Knowing what I and others are thinking’); those who reported not having any thoughts but a strong emotional reaction (‘It is just a feeling’); those who reported ‘Finding it hard to explain’ what they were thinking, and; those who were unclear about cognitions (‘Not knowing’). Theme three, ‘Managing social fears’ contained two related subthemes (‘Wanting to get out of here’ and ‘Trying to come over as likeable’) and concerned *how* the children try to manage their social fears and associated anxiety. Relationships between the themes were evident. Anonymized (through pseudonyms) participant comments are provided below to evidence the findings.

### Theme one: Discomfort being the centre of attention

#### I will do something wrong

All of the children endorsed the view that social situations were threatening, and experienced great discomfort when they perceived they were the centre of attention.

When asked to report on what they were thinking and if there was anything they were concerned about, children typically expressed concerns about their own social performance:

CC (age 11) …I will say something wrong.

DD (age 12) I will make a fool of myself.

Notably, two of the older children who reported fears about doing something embarrassing, also spoke about ‘not knowing’ what to do in social situations, indicating negative beliefs about their ability to manage social interactions:

AA (age 12) Usually in [social] situations, I'm meant to do something, and I'm scared I'm going to fail to do it…I don't know what to do.

DD (age 12) …all I know is that when people are looking at me I just get ‘oh what do I do?’ Do I stand still, or do I move…I don't know.

#### I am being judged

In addition to being concerned about their own social performance, it was evident that many participants perceived that the children in the pre‐recorded audience were judging them negatively. For example, both KK (age 10) and HH (age 12) said the others found them ‘boring’ and DD (age 12) said the children were thinking ‘he's a fool’. Notably, none of the children reported that the children in the pre‐recorded audience had positive thoughts about them and/or liked them.

HH (age 12) They wouldn't want to make friends with me

Furthermore, although the children in the recording were neutral in their responses and showed no signs of laughing, some participants perceived that they were being laughed at:

JJ (age 11) There were these three girls laughing, and I thought they were laughing at me.

Whilst most children had specific ideas about what the children in the audience were thinking of them, others were less sure. For example, FF (age 11) said she was ‘not sure’ what the children were thinking, but her non‐verbal behaviour (e.g., avoiding looking at the screen, appearing distressed) and further questioning indicated it was something negative. Notably, FF's explanation here suggests that she is aware that her negative predictions may not necessarily be true:

FF (age 11) …I'm not sure what they are thinking, I kind of think for them, like make up what they are thinking.

Interviewer And is that negative?

FF (age 11) Yeah.

#### Sense of being stared at

Several children commented that they perceived the other children in the audience to be staring at them.

KK (age 10) …they are staring at me in like… a like… a weird way… Eh… I feel like different because like they are staring at me.

It was noticeable that KK reported that the sense of being stared at made her feel ‘different’. The other children used a range of words that suggested this feeling of being stared at had a significant impact on how they felt, for example ‘overwhelmed’ (II, age 12); ‘confused’ (DD, age 12) and ‘freaked out’ (FF; age 11). Notably, when participants were interviewed again 1 week later to specifically ask them about post‐event processing, FF was the only participant that appeared to engage in this behaviour and the thought content was focused on being looked at and the experience of embarrassment:

Interviewer Over the last week, did you have any thoughts about the video?

FF (age 11) I spoke to mom [after the session] and thought they were all looking at me.

Interviewer Any other thoughts you have?

FF (age 11) Like… embarrassing one. And how they are all staring.

For two of the oldest children, the sense of being stared at did trigger negative images and when asked to describe how they ‘saw’ themselves in the image they said:

II (age 12) …like red, and hot and ehm… just ehm… sweaty.

DD (age 12) Bigger than I am… looking like an idiot? Just standing out from everyone else. Kind of behind everyone else.

### Theme two: (Lack of) awareness of cognitions

#### Knowing what I and others are thinking

Some children engaged well with questions about their cognitions throughout the interview and were able to respond quickly with thoughts about being ‘unlikeable’ (KK, age 10) or give more detailed answers describing how different children in the pre‐recorded audience had different views about them:

KK (age 10) She [pointing at a girl in the pre‐recorded audience] might be thinking ‘she is not very nice’.

Interviewer What about the boys, what are they thinking?

KK That one looks a bit nervous. And the other one is really serious….He could be worried and doesn't want to show it…

Interviewer What about these here [pointing at other children in the audience]? KK

They might not like me, they might start laughing at me.

#### It is just a feeling

Although many participants engaged well with questions about their cognitions, others could not describe specific thoughts or fears associated with anxious feelings. For example, LL (age 8) said he was worried about feeling ‘nervous’ but answered ‘no’ when asked whether there was anything specific he was worried about. Similarly, AA (age 12) said he often did not know what he was afraid of and described judging social situations on how they made him feel:

Interviewer Okay, so I can see you're feeling anxious now. And I can see that you're not looking at them. Is there a reason for that?

AA (age 12) I just don't want to see them.

Interviewer Can I ask why?

AA It's just a feeling.

Probing encouraged some children to express thoughts, but not everyone. For example, despite being visibly very anxious and reporting that her tummy hurt, BB (age 11) whispered when asked what she was thinking:

BB (age 11) I don't have any thoughts.

It was noticeable that both participants BB and AA showed a particularly tense body posture during the task—appearing as if they were frozen. Indeed, AA (age 12) specifically commented that he ‘usually just stand[s] there, frozen’ when asked how he normally felt whilst standing in front of his peers at school (e.g., reading in front of the class).

#### Finding it hard to explain

Others appeared to imply that their social anxiety was linked to specific negative cognitions but found it hard to explain what they were:

Interviewer Anything that you are anxious about?

DD (age 12) ehmm…Sort of.

Interviewer Sort of. Ok. Can you tell me what that is?

DD Eh…no it is hard to explain.

Later on, DD reported that this is how he normally felt before and after social interactions—that is, anxious and worried, but finding it hard to explain why.

#### Not knowing

Other children (particularly younger participants) commonly said ‘I don't know’ or ‘I'm not sure’ when asked about their cognitions. For these children, it was unclear whether they felt anxious without a clear associated negative cognition or whether they could not explain or articulate what they were thinking. For example, one child said loudly: ‘No, no no…’ (LL, age 8) when the pre‐recorded audience appeared on the wall and ran to hide behind the interviewer and asked for the video to be turned off. When asked what it was that was making him anxious, he replied: ‘I don't know’. Furthermore, despite our efforts to try to explain concepts in age‐appropriate ways and give participants opportunities to express imagery in a number of ways (e.g., through drawing)—most participants found it hard to engage with discussions about imagery and looked blank when asked about this concept.

### Theme three: Managing social fears

#### Wanting to get out of here

The children identified several strategies that they used to manage their social fears and associated anxiety. Unsurprisingly, most children reported that they typically tried to avoid social interactions:

LL (age 12) … I will try to look busy, like go on my phone, try to look away so I have to talk to someone.

Within‐situation avoidance behaviours, such as avoidance of eye contact, were also commonly reported across the age range:

GG (age 10) …if I feel nervous, I don't like to look at people when I'm talking. I actually look at something that is not looking back at me.

When avoidance was not possible, children commonly endorsed attempting to escape from social situations:

AA (age 12) I just want to get out and stop focusing on how I say things and look at other people.

#### Trying to come over as likeable

When children were asked specifically about other cognitive and behavioural safety‐seeking behaviours (i.e., if there was anything they were doing to help themselves feel less anxious either at that moment or in other social situations where they could not avoid or escape), the younger children struggled to engage with the conversation, whereas the older children were more likely to provide examples. One child commented specifically about trying to ‘look normal’ (DD, age 12) but was unable to say what that involved. Another child said she tried to act friendly to make herself feel safer and less anxious:

FF (age 11) …[I] try to make contact, like nice contact…and, just smile and try to be nice… I try to make them think I'm a nice person.

Two (older) children reported engaging in cognitive safety‐seeking behaviours, specifically preparing what to say in advance:

II (age 12) I'm thinking of what I say before I say stuff.

Children also appeared to describe engaging in what appeared like soothing behaviours, such as ‘play with a string’ (FF, age 11) or ‘[Have] my hands in my pockets to mess around’ (DD, age 12) to distract themselves from their social anxiety.

## DISCUSSION

This is the first qualitative study that we are aware of that is focussed upon ‘in the moment’ social experiences and perceptions of pre‐adolescent children with SAD, in order to inform a greater understanding of the maintenance of the disorder. Discomfort being the centre of attention was evident for all the children, and in many cases, this was driven by the negative belief/s that they would do something wrong, be judged and/or stared at by others. This led to a variety of emotional and behavioural reactions intended to deal with the perception of threat, some of which have been described in the previous literature with socially anxious children and some which have not. More specifically, as noted in previous research recruiting pre‐adolescent children with SAD (e.g., Alkozei et al., 2014; Kley et al., 2012; Spence et al., 1999), there was evidence that the children's negative beliefs reflected a sense of social threat, ambiguous neutral stimuli (e.g., the neutral facial expressions of children in the pre‐recorded audience) were interpreted in an overly negative fashion, and within‐situation avoidance was seen as a ‘helpful’ strategy to avert feared outcomes. Notably, among the *older* children in the study, potential maintenance factors were described that have not previously been systematically examined in the (pre‐adolescent) child‐focused literature on SAD; including the use of safety‐seeking behaviours, post‐event processing and experience of negative imagery, which appeared to reinforce children's negative belief/s about how they appeared to others. In contrast to the adult CBT models (Clark & Wells, 1995; Rapee & Heimberg, 1997), children's narratives did not indicate that they experienced diffused body perception or a felt sense (i.e., reported using their internal experiences as evidence that others were judging them or described a compelling feeling that encapsulated their social fear). For example, none of the children reported feeling embarrassed, shaking, sweating and/or stupid and interpreting such feelings as evidence that others saw they were embarrassed, and noticed that they were shaking and sweating and/or felt they came across as stupid.

At present, the most frequently used psychological treatment for childhood SAD is multi‐disorder‐focused CBT (e.g., Kendall & Hedtke, 2006). This typically includes a core combination of challenging negative thoughts and behavioural exposure (often to target avoidance). Thus, whilst a number of mechanisms identified here are directly targeted in multi‐disorder‐focused CBT, others (in particular safety‐seeking behaviours, negative imagery and self‐focused attention) are not explicitly addressed. Furthermore, even though many of the main mechanisms that are targeted in multi‐disorder CBT do seem to apply for pre‐adolescent children with social anxiety, they appear to apply to a greater or lesser extent across individuals. For example, there was variation in children's awareness of their negative cognitions. Whilst some children had no difficulty articulating their social fears, other children reported no links between their social anxiety and negative cognitions, or were unable to explain what they were thinking, or reported not having any thoughts at all. In addition, it seems likely that there are developmental influences on which maintenance mechanisms apply at which point in time as only older children in the sample reported safety‐seeking behaviours, negative imagery and post‐event processing, indicating that more disorder‐specific CBT interventions may apply in early adolescence. Human brain development undergoes vast developmental changes between childhood and adulthood which affect key social and cognitive capabilities such as self‐awareness, peer influence and sensitivity to social rejection (Kilford et al., 2016). It is likely that the development of these social and cognitive capabilities influences the presentation and timing of several psychological mechanisms. In support of this, we note that older children in this study were more likely to engage in sophisticated safety‐seeking behaviours like impression management than younger participants, indicating that this may potentially develop with age. Notably, a recent study (Evans, Chiu, et al., 2021) reported age‐related effects on the use of particular safety‐seeking behaviours. That is, older adolescents engaged more frequently than younger adolescents in safety‐seeking behaviours (in particular, impression management behaviours) that are considered to require complex social cognitive skills. It should also be noted that the two subthemes identified in this study (i.e., ‘Wanting to get out of here’ and ‘Trying to be likeable’) that focused on children's safety‐seeking behaviours, align with findings from factor analytic studies with adults with SAD (e.g., Gray et al., 2019; Plasencia et al., 2011) and more recently adolescents with SAD (Evans, Chiu, et al., 2021; Evans, Clark et al., 2021) that suggest that safety‐seeking behaviours in SAD can be subdivided into two broad categories: avoidance and impression management strategies. We also note that only two participants (whom both were among the oldest participants in the study) reported experiencing distorted negative self‐imagery. It is possible that the lack of negative imagery in our sample results from limited experiences of distressing (traumatic) social events (e.g., bullying, being humiliated) among our participants, as such events have been linked to the development of negative imagery in adults with SAD (Hackmann et al., 2000). However, it is also possible that this finding reflects developmental variation in the association between cognition and affect. Specifically, whilst it is well‐established that pre‐adolescent children experience and use imagery (e.g., imaginary play; Tobin et al., 2013), it has been argued that the relationship between social anxiety and imagery likely changes during the course of childhood and adolescence as individual's competence in mental imagery (e.g., ability to generate and reflect on an image) is likely to alter with age (Heyes et al., 2013). Clearly, future studies should take a developmentally informed approach to help us to understand the nature of the association between these factors and social anxiety in childhood and how that may change through development.

Given the preliminary nature of the findings of this study, any implications for how treatment may be adapted in order to make it more targeted and efficient must be extremely tentative and further research is needed to make any strong recommendations. However, the preliminary findings highlight the importance of understanding from the beginning of treatment what maintenance mechanisms apply to each child, followed by the delivery of treatment in a flexible manner where specific treatment components are added or modified to meet the individual needs of the child. For example, experiencing negative imagery and engaging in safety‐seeking behaviours—two processes described by DD—are typically not targeted in multi‐disorder‐focused CBT. Children like DD may benefit from the use of video feedback—a core technique for addressing safety‐seeking behaviours in cognitive therapy for adults with SAD (Warnock‐Parkes et al., 2017), that has recently shown promise for adolescents (Leigh & Clark, 2018). Furthermore, children that are concerned about exhibiting flaws in social competence and coming across as anxious may benefit from treatment interventions that target such concerns and have been found to be helpful for adults with SAD (see Moscovitch, 2009). In contrast, for those children who appeared less aware of their cognitions, persisting with questioning them about their negative thoughts may be disheartening and unhelpful, whereas focusing mainly on behavioural exposure may be more appropriate. However, there may also be additional (yet unknown) processes contributing to the maintenance of social anxiety in these children and this warrants further examination.

One of the significant strengths of qualitative studies is that they encourage theory development from the lived experiences of study participants (Braun & Clarke, 2013). Indeed, findings from qualitative studies investigating the lived experiences of adults with SAD have resulted in a better understanding of the nature and maintenance of social anxiety in adult populations and supported the development of effective treatment interventions (e.g., Hackmann et al., 2000). That so many participants were able to give detailed descriptions of their experiences suggests that, despite their social anxiety, there is merit in sensitively using social stress tasks to explore how children with SAD experience and interpret social events. Nonetheless, our findings should be considered in light of their limitations. First, interviewing children presents several challenges including communicating about concepts in age‐appropriate ways and making sure they can express their views and ideas (Gibson, 2012). Despite careful use of language and the use of several items and prompts to make it easier for study participant's (who, given their presenting problem, find social interactions particularly difficult) to express themselves, not all children were able to provide in depth, rich responses in the interviews. It is also important to note that some participants may have found the dual nature of the social stress task (i.e., watching a video whilst also answering questions) complex and difficult to process, which may have had an impact on their ability to answer the study's questions. This was a novel procedure and further examination is required, however, that so many children were able to give detailed descriptions of their experiences, provides evidence of ecological validity and, demonstrates that, despite the difficulties, there is merit in asking children with SAD to participate in research of this kind. Second, the study is qualitative and so statistical‐probabilistic generalization to the population is not possible. Instead, the goal of qualitative research is to provide a rich, contextualized understanding of children's experience of social anxiety disorder in order to extend the transferability of the findings beyond the study sample and make analytic generalizations to theory (Polit & Beck, 2010). Third, it is likely that there may be other potential maintenance mechanisms that we did not identify and explore within this study. For example, peer interactions, such as low peer acceptance and/or victimization, and particular parental practices may play a role in maintaining social anxiety in children (Halldorsson & Creswell, 2017). Furthermore, although the topic guide was modified iteratively to incorporate new information and focus progressively on themes, it was influenced by two particular cognitive models of adult SAD (Clark & Wells, 1995; Rapee & Heimberg, 1997). It is important to note that other models of adult SAD have been published over recent decades that include distinct components. For example, Hofmann (2007) emphasizes the role of ‘low perceived emotional control’ and ‘poorly defined social goals’, and Moscovitch (2009) argues that particular self‐relevant concerns play a role in maintaining social anxiety (i.e., individuals with social anxiety are concerned about exhibiting flaws in social competence, showing visible signs of anxiety and exhibiting flaws in physical appearance). Future studies would benefit from examining whether these factors also play a role in maintaining childhood social anxiety. Notably, in support of Moscovitch (2009) model, some children in this study expressed concerns about their social competence and (to some extent) coming across as anxious; although notably they did not specifically describe concerns about their physical appearance. Fourth, although our sample, with its high levels of comorbidity, is clinically representative (Kendall et al., 2010; Waite & Creswell, 2014), it is not clear to what extent children's responses reflect their experience of social anxiety disorder specifically. Another key limitation is that the participants were all White British. Although reflecting the typical patient receiving treatment/assessment in the setting in which they were recruited, future research should explore ‘in the moment’ social experiences of children from more diverse backgrounds as there is evidence to suggest that culture may influence the presentation and expression of anxiety problems in children (Varela et al., 2019) and adults (Hofmann et al., 2010). Finally, and critically, although the whole research team took part in shaping the data and comprised researchers from different backgrounds, three authors have expertise in CBT, and this experience is likely to have had an effect on the interpretation of the data in this study.

## CONCLUSIONS

This analysis of the ‘in the moment’ social experiences of children with SAD highlights the varied presentation of childhood SAD which should be considered when undertaking clinical work with children with SAD. There appear to be differences in which maintenance mechanisms children with SAD endorse and developmental factors are likely to influence when specific mechanisms come ‘online’. In line with SAD treatment development procedures within the adult field (Clark, 2004), further studies are now needed to (i) experimentally manipulate the psychological factors identified in the current study to examine which factors truly play a role in maintaining childhood SAD; (ii) use those findings to develop specific treatment strategies to target the identified maintenance mechanisms; and (iii) develop instruments that allow us to identify which mechanism (individual) children with SAD express and use the same instruments to guide the focus of treatment.

## AUTHOR CONTRIBUTIONS

**Brynjar Halldorsson:** Conceptualization; data curation; formal analysis; investigation; methodology; project administration; resources; writing – original draft; writing – review and editing. **Polly Waite:** Formal analysis; writing – original draft; writing – review and editing. **Kate Harvey:** Formal analysis; writing – original draft; writing – review and editing. **Samantha Pearcey:** Data curation; writing – original draft; writing – review and editing. **Cathy Creswell:** Conceptualization; formal analysis; investigation; methodology; supervision; writing – original draft; writing – review and editing.

## CONFLICT OF INTEREST

All authors declare no conflict of interest.

## Supporting information


Appendix S1

## Data Availability

The data that support the findings of this study are available on request from the corresponding author. The data are not publicly available due to privacy or ethical restrictions.
