# Supplementary material for: In the moment social experiences and perceptions of children with social anxiety disorder: A qualitative study
Source: Br J Clin Psychol. 2022 Oct 10;62(1):53–69. doi: 10.1111/bjc.12393 (PMC10091697; doi:10.1111/bjc.12393)
Supplement: Supplementary file 1 — Appendix S1 [file BJC-62-53-s001.docx]

**Supplementary material**

| **Representative Interview Guide – Examples of: props, how concepts were introduced and interview questions.** |
| --- |
| **Anticipatory processing**  *Note:* *At first, the video of the pre-recorded audience was turned off, as the first interview questions focused on what the participants experienced prior to a social event (e.g. did they feel anxious, what were they thinking, was there anything they were worried about). Once this section of the interview was completed, the video was turned on and the remaining sections of the interview were completed with the video running and the child standing in front of the screen.*  Props: Picture of the pre-recorded audience to show participants what to expect  Introducing concept: *So, as I briefly explained earlier, soon you will be answering some questions whilst standing in front of other children (who were video recorded).*   - *Can you tell me how you feel about doing this?* - *What do you expect to happen?* - *What kinds of thoughts do you have?* - *Is there anything specific that you are worried about?* |
| **Negative thoughts**  Props: Thought bubbles, pens, paper for writing, list of common negative and positive thoughts  Introducing concept: *I am now going to turn on the video recording. I would like you to imagine that this is real, as if it is happening to you right now. What I really want to do is get an idea of what you are thinking [we can add some thought bubbles to the screen/wall if you think that would be helpful].*   - *Can I check what you are thinking right now?* - *Is there anything you are worried about?* - *What is the worst that you think can happen?* - *What do you think the other children are thinking?* |
| **Anxious feelings**  Props: Paper, coloured pens, cartoon characters showing common bodily symptoms of anxiety (e.g. tummy ache, sweating, heart beating fast).  Introducing concept: *So, how does having these thoughts make you feel?*   - *How do you feel right now?* - *If you had to describe how you feel, what words would you use?* - *Do you notice any sensations in your body?* |
| **Self-focused attention**  Props: Flashlight, cartoon picture showing difference between first person and third person view; paper to draw images, coloured pens.  Introducing concept: *See this flashlight here. For some people, it is sometimes like their attention is like this [point flashlight towards self] and for others it is like their attention is here [point flashlight at screen]*   1. **Focus of attention:**  - Where is your attention? Are you feeling self-conscious? - Are you focusing on yourself and how you think you may appear?  1. **Contents of self-image**   Introducing concept: *Sometimes people have pictures or even like a brief video playing of how they think they look and how other people see them. Sometimes these images are really negative, like someone who is afraid of blushing might have a picture of themselves looking red like that sofa [point to red sofa in the room]?*   - *Do you ever experience that?* - *Do you have an image/video of yourself?* - *Do you have a picture in your mind of what you look like?* |
| **Safety-seeking behaviours**  Props: Magic wand [toy], images of children engaging in safety-seeking behaviours, list of common safety-seeking behaviours  Introducing concept: *Some children do certain things when they are in social situations, like think special things, carry a toy, make sure to have their mom… is there anything that you are doing now to make you feel less worried or less anxious?*   - *Some children do certain things when they are in social situations – like think special things, carry a toy, make sure to have someone they trust (e.g. mum). Is there anything that you are doing now to make you feel less worried.* - *When you thought [child’s fear] was happening, did you do anything to try to stop it from happening?* |
| **Post-event processing**  Introducing concept: *Do you remember the last time we met? Can you tell me a bit about what we were doing?*   - *How did you find the session?* - *After the session was over did you think about it much? Why?* - *For how long?* - *What were you thinking/worried about?* - *How did these thoughts make you feel?* - *Did you find it hard not to think about it? Why?* |
